# Comprehensive Overview of the Effects of *Amaranthus* and *Abelmoschus esculentus* on Markers of Oxidative Stress in Diabetes Mellitus

**DOI:** 10.3390/life13091830

**Published:** 2023-08-29

**Authors:** Wendy N. Phoswa, Kabelo Mokgalaboni

**Affiliations:** Department of Life and Consumer Sciences, University of South Africa (UNISA), Science Campus, Private Bag X6, Florida, Roodepoort 1710, South Africa; mokgak@unisa.ac.za

**Keywords:** antioxidants, diabetes mellitus, *Amaranthus*, *Abelmoschus esculentus*, oxidative stress

## Abstract

The use of medicinal plants in the management of diabetes mellitus (DM) is extensively reported. However, there is still very limited information on the role of these plants as markers of oxidative stress in DM. This current review evaluated the effect of *Amaranthus spinosus*, *Amaranthus hybridus,* and *Abelmoschus esculentus* on markers of oxidative stress in rodent models of DM. Current findings indicate that these plants have the potential to reduce prominent markers of oxidative stress, such as serum malondialdehyde and thiobarbituric acid-reactive substances, while increasing enzymes that act as antioxidants, such as superoxide dismutase, catalase, glutathione, and glutathione peroxidase. This may reduce reactive oxygen species and further ameliorate oxidative stress in DM. Although the potential benefits of these plants are acknowledged in rodent models, there is still a lack of evidence showing their efficacy against oxidative stress in diabetic patients. Therefore, we recommend future clinical studies in DM populations, particularly in Africa, to evaluate the potential effects of these plants. Such studies would contribute to enhancing our understanding of the significance of incorporating these plants into dietary practices for the prevention and management of DM.

## 1. Introduction

Diabetes mellitus (DM) is a chronic, life-threatening disease that has caused more than 6.7 million deaths worldwide [1]. This condition affects approximately 537 million adults (20–79 years old) [1]. According to the International Diabetes Federation, the number of people living with diabetes is predicted to reach 643 million by 2030 [1]. There are three commonly known types of DM: type 1 diabetes mellitus (T1DM), type 2 diabetes mellitus (T2DM), and gestational diabetes mellitus (GDM). T2DM is considered the most common, and it affects [2] at least 95% of the diabetic population [3]. In 2021, the prevalence of T2DM was reported to be 10.5% [4], and most of the population is from low- and middle-income countries (LMICs) [1].

Diabetic patients in LMICs face many challenges, which include a lack of awareness and knowledge about the disease, difficulty accessing health care systems, including medications, and inadequate diabetes management strategies, which likely result from a poor socio-economic background [5]. Biological risk factors associated with DM include older age, increased body mass index (BMI), obesity, stress, physical inactivity, and chronic inflammation due to other infectious diseases [6,7,8]. DM is associated with health complications such as cardiovascular diseases, kidney diseases, vision impairment, and neurological conditions [9]. Oxidative stress has been reported to play a major role in the pathophysiology of DM-related complications [10].

In 2021, it was documented that DM caused at least 966 billion USD in health expenditure, with 9% of total spending on adults [1]. Low- and middle-income countries already have overwhelming health burdens resulting from other common diseases such as tuberculosis and the human immunodeficiency virus [11]. Therefore, healthcare systems in place must implement efficacious medicines that are less toxic and cost-effective in the management of DM.

While medical and pharmacological drugs are currently available for managing DM, these are still associated with severe side effects in different individuals, increased complications, and a rising mortality rate in DM. For instance, using sodium-glucose cotransporter 2 inhibitors increases the risk of hypotension, diabetic ketoacidosis, kidney injury, and bone fractures [12]. Regrettably, prolonged use of glucophage is linked to cobalamin deficiency, increasing the risk of additional complications, including anemia, in T2DM patients [13]. Given the mentioned drawbacks of pharmaceutical medications, there has been a burgeoning interest in the utilization of functional foods and herbal remedies for the treatment and management of DM. This interest is partly attributable to their inherent properties. Numerous studies have highlighted the antioxidant characteristics of medicinal plants in the treatment and management of DM. 

For instance, our team recently found the potential beneficial effects of *Corchorus olitorius* and curcumin in a rodent model of obesity and DM and T2DM, respectively [14,15].

Interestingly, functional fruits have also demonstrated potential benefits, especially on oxidative stress in DM [16]. Although there are rising calls for more research support for medicinal plant use in the treatment and management of DM [11], there is still limited clinical evidence to support their efficacy, especially in DM. Moreover, evidence from previous preclinical studies has not focused on common markers of oxidative stress. In this study, we aim to gather evidence from preclinical studies evaluating the effect of *Amaranthus hybridus*, *Amaranthus spinosus*, and *Abelmoschus esculentus* in DM primarily due to their beneficial properties and safety profile [17], with the main focus on various biomarkers of oxidative stress. Therefore, this review will highlight and document the potential benefits of these selected medicinal plants in DM.

## 2. Oxidative Stress and Diabetes Mellitus

Oxidative stress occurs as a result of an imbalance between the production and clearance of reactive oxygen species (ROS) [18] and contributes to the pathogenesis and pathophysiology of DM [19,20]. DM is a metabolic disorder characterized by increased blood glucose levels resulting from insulin resistance and impaired insulin secretion [21].

In DM, several factors contribute to oxidative stress, including hyperglycemia, dyslipidemia, insulin resistance, and inflammation. Hyperglycemia and hyperlipidemia can lead to increased cellular oxidative stress through mitochondrial electron leak or incomplete fatty acid oxidation, the formation of advanced glycation end products (AGEs), lipid hydroperoxides, and induced free fatty acids (FFA), diacylglycerol (DAG), and ceramides. Lipid peroxidation has been identified as one factor that leads to DM development [22,23]. This occurs when there is uncontrolled high blood glucose and free fatty acids, which in turn activate DAG and protein kinase C (PKC) [24,25,26]. Activation of PKC induces the inflammatory response by promoting the secretion of endothelin 1 (ET-1), vascular cell adhesion molecule (VCAM-1), intercellular adhesion molecule (ICAM-1), nuclear factor kappa-light chain enhancer of activated β cells (NF-κβ), and NADPH oxidase [27,28,29]. Notably, NADPH oxidase activation mediates ROS generation through superoxide [30,31]. Excessive ROS production damages cells, resulting in a pronounced inflammatory response [32]. Hence, it is comprehensible why diabetes is frequently linked to inflammation [32,33,34,35]. Conversely, catalase (CAT), an active enzyme, functions as an antioxidant by catalyzing the conversion of hydrogen peroxide into water and oxygen [36]. Nevertheless, diminished CAT activity results in oxidative stress in the pancreatic beta cells, which contain numerous mitochondria. This excess production of reactive oxygen species (ROS) ultimately leads to dysfunction in β-cells and the onset of diabetes [18]. Therefore, this would subject the cells or organs to oxidative stress by allowing the accumulation of harmful oxidants and free radicals. Reactive oxygen species can also increase insulin resistance, leading to further hyperglycemia [37,38,39,40,41]. This occurs when caloric intake exceeds energy expenditure, thereby causing an increase in citric acid cycle activity. This subsequently leads to excess mitochondrial NADH (mNADH) and ROS [42]. Inflammation, which is common in DM, can also contribute to oxidative stress by activating immune cells that produce ROS [43]. Several studies have indicated that oxidative stress can contribute to the development of diabetic complications such as neuropathy, retinopathy, and nephropathy [43,44,45,46]. Therefore, controlling oxidative stress may be an important DM management strategy [44]. This can be achieved through lifestyle modifications such as regular exercise, a healthy diet, and smoking cessation [47]. Antioxidant supplements such as vitamin C, vitamin E, and alpha-lipoic acid may also be beneficial in reducing oxidative stress [48]. Some medicinal plants, such as *Amaranthus spinosus*, *Amaranthus hybridus*, and *Abelmoschus esculentus*, have been shown to have antioxidant effects.

## 3. *Amaranthus* Species

*Amaranthus*, a herbaceous plant native to Central America, has been cultivated for centuries due to its valuable properties [49]. It has spread to various nations, successfully established itself, and naturalized in numerous regions across the globe [50]. In Africa, it is esteemed as a traditional food plant, thus providing a valuable source of nutrition. All parts of the plant, including seeds, roots, leaves, and stems, are recognized for their edible and medicinal attributes [51]. In addition, *Amaranthus* is affordable and cost-effective [52], making it significant in improving nutrition, ensuring food security, and alleviating poverty, especially in low- and middle-income countries [53]. *Amaranthus* has been recognized as a superfood, making it an interesting plant for further exploration in research [54]. Among the various *Amaranthus* species, the common ones include *Amaranthus thunbergii*, *A. greazican*, *A. deflexus*, *A. hypochondriacus*, *A. viridis*, *A. spinosus*, and *A. hybridus*. This pivotal plant possesses an abundance of vital nutrients, making it a rich source of essential nutrients, including vitamins and minerals [54]. These micronutrients have been extensively studied for their crucial role in promoting optimal well-being. More interestingly, *Amaranthus* has been reported to possess various compounds, including amino acids such as lysine, arginine, histidine, leucine, cysteine, phenylalanine, isoleucine, valine, threonine, and methionine [55].

**Figure 1:**
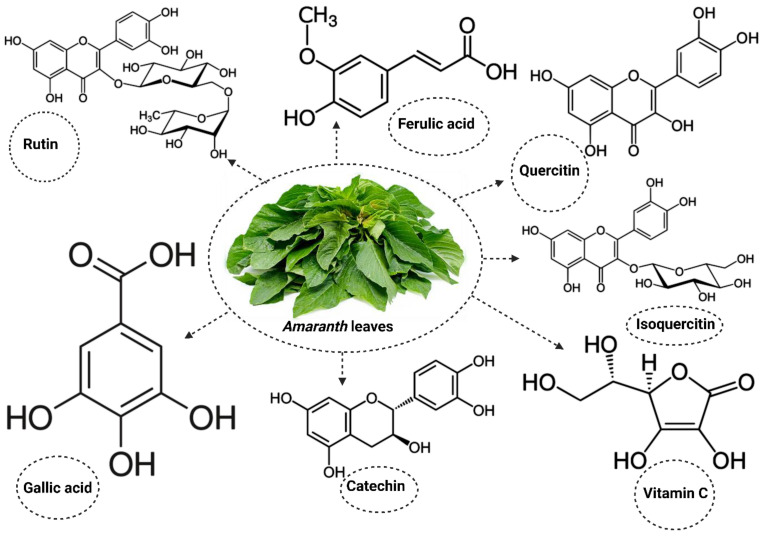
Some of the active compounds present in *Amaranthus*.

The *Amaranthus* plant also contains important active compounds that promote its activities [56,57,58] (Figure 1). The use of *Amaranthus* in the public interest extends beyond its nutritional benefits to its therapeutic properties, especially in the management of cholesterol and blood glucose levels in DM [59,60]. While many studies reporting on the therapeutic properties of *Amaranthus* in DM have focused on inflammatory markers [61,62,63], only a limited number of studies have explored its benefits in alleviating oxidative stress in DM. The present study will review studies reporting the potential benefits of *Amaranthus spinosus* and *hybridus* on markers of oxidative stress in DM. This will help to improve current knowledge on the importance of consuming these plants, especially in diabetic individuals, in order to manage the condition and in non-diabetic individuals to prevent the risk of developing DM.

### 3.1. Amaranthus spinosus

**Figure 2:**
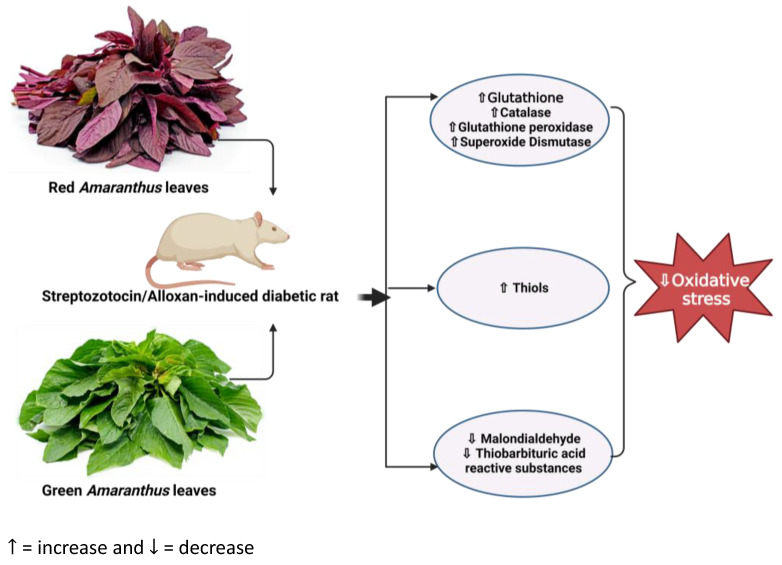
Overview showing the impact of *Amaranthus* on oxidative stress in diabetic rats. Administration of *Amaranthus* in diabetic rats ameliorates oxidative stress by reducing malondialdehydes and thiobarbituric acid while increasing the activity of antioxidant enzymes such as glutathione, glutathione peroxidase, catalase, and superoxide dismutase. (https://www.seeds-gallery.eu/9135-large_default/amaranth-red-garnet-seeds-Amaranthus-tricolor.jpg (accessed on 25 July 2023), https://specialtyproduce.com/produce/Green_Amaranth_12831.php (accessed on 25 July 2023).

As presented in Table 1, *Amaranthus spinosus* has been shown to alleviate oxidative stress. *Amaranthus spinosus* has been shown to reduce hyperglycemia-associated oxidative stress significantly in rodent models of obesity [64]. A study by Kumar et al. (2011) reported that methanol extract of *Amaranthus spinosus* at a dose of 200 and 400 mg/kg for 15 days had antioxidant effects against DM [65]. This was demonstrated by a reduction in the serum level of malondialdehyde (MDA) concomitant with the increased activity of enzymes that act as antioxidants, such as glutathione (GSH) and catalase (CAT), in alloxan-induced diabetic rats. Furthermore, the same study showed an increase in total thiols. Similar findings were observed by Mishra et al. (2012), whereby *Amaranthus spinosus* leaf extract (ASEt) at doses of 250 and 500 mg/kg for 21 days reduced oxidative stress and improved pancreatic cell function in diabetic rats [66]. These positive impacts were demonstrated by a significant increase in superoxide dismutase (SOD), CAT, GSH, and glutathione peroxidase (GPx). An increase in antioxidant enzymes observed after *Amaranthus* treatment shows its potential as an antioxidant remedy (Figure 2). Therefore, this suggests that this plant could play an important role in alleviating oxidative stress.

### 3.2. Amaranthus hybridus

Similarly, *Amaranthus hybridus* has been reported to have anti-oxidative stress effects in DM [67]. *Amaranthus hybridus* ethanol leaf extract (AHELE) has been identified to have a nephron protective effect against oxidative damage in streptozotocin (STZ)-induced diabetic rats [68]. In a study by Balasubramanian et al. (2016), this was indicated by a significant reduction in the marker of lipid peroxidation, thiobarbituric acid reactive substances (TBARS) (*p* < 0.001), and a significant increase in antioxidant markers such as superoxide dismutase (SOD) (*p* < 0.001) and CAT (*p* < 0.01). In support of their findings, they also discovered that AHELE possessed both nephroprotective and hepatoprotective effects by reducing the levels of MDA in the liver and kidneys of STZ-induced diabetic rats [68]. Therefore, the evidence from rat models of diabetes induced by STZ or alloxan indicates the potential of the *Amaranthus* plant as an anti-oxidative stress agent (Figure 2, Table 1). MDA is an end-product of fatty acid peroxidation [69], and its high level is an indication of lipid peroxidation [70]. During lipid peroxidation, lipids are degraded in the cell membrane, thus leading to cell damage [69,71]. Lipid peroxidation has been identified as one of the factors leading to the development of DM [22,23].

## 4. *Abelmoschus esculentus*

**Figure 3:**
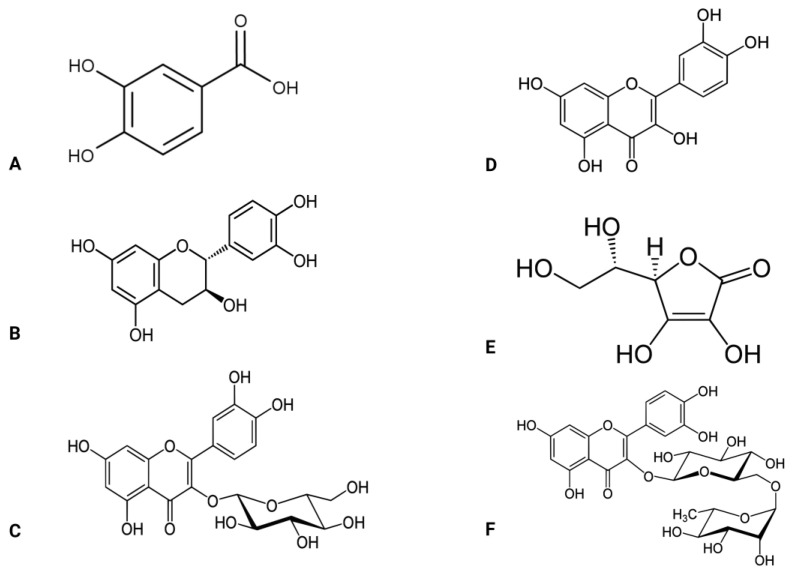
Active compounds found in the *Abelmoschus esculentus* plant. (**A**) protocatechuic acid, (**B**,**C**) catechin, (**D**) quercetin, (**E**) vitamin C, (**F**) rutin.

*Abelmoschus esculentus* L. is also known as okra and belongs to the Malvaceae plant family. Although this plant is found in Africa, it is widely distributed in Asia, America, and Southern Europe [72]. The fruit, seeds, roots, leaves, flowers, and pods of *Abelmoschus esculentus* contain vital bioactive chemicals that contribute to this plant’s beneficial effects [73,74] (Figure 3). For example, the seed contains oligomeric catechins and flavonol derivatives [74,75,76]. The root contains carbohydrates and flavonol glycosides [77], while the leaves contain minerals, tannins, and flavonol glycosides [78]. The pod contains carotene, folic acid, thiamine, riboflavin, protein, fiber, calcium, iron, zinc, niacin, vitamin C, oxalic acid, and amino acids [79,80,81]. These compounds mediate the various functions that *Abelmoschus esculentus* possesses. Such potential benefits include but are not limited to anti-hyperglycemic, anti-inflammatory, and antioxidant effects [82,83,84].

### 4.1. Antioxidant Effect of Abelmoschus esculentus

The antioxidant properties of *Abelmoschus esculentus* have been revealed by previous research [83,84,85,86], and this is attributable mainly to its active compounds, such as polyphenols and flavonoids [87] (Figure 3). Polyphenols mediate antioxidant activity by reducing MDA and increasing SOD, GPx, and catalase activity [77,78,82,84,85,86,87,88,89,90,91,92,93]. Some of the active polyphenol compounds include isoquercetin, quercetin, quercetin-3-O-gentiobioside, quercetin-3-O-glucoside, protocatechuic acid, and rutin. All these phenolic compounds exhibit free radical scavenging and ferric-reducing properties [92] and further inhibit the activities of α-glucosidase and α-amylase [94]. Specifically, the *Abelmoschus esculentus* seeds are excellent sources of phenols, including procyanidin B1 and B2, which facilitate the free radical scavenging activities of 1,1-diphenyl-2-picrylhydrazyl (DPPH) and 2,2′-casino-bis (3-ethylbenzothi azoline-6-sulfonic acid (ABTS) [90,95,96].

#### 4.1.1. Effect of *Abelmoschus esculentus* on Oxidative Stress in Animal Models of Diabetes Mellitus

Mice and rats have been used for decades to mimic DM observed in humans, primarily to explore the beneficial effects, toxicity, and desirable doses of different compounds against various metabolic disorders [97]. Oxidative stress is implicated in the progression of insulin resistance into DM due to the increased production of free radical molecules. These ROS molecules (hydrogen peroxide, superoxide anion, and hydroxyl radicals) are generated by the partial reduction of oxygen molecules [98]. However, when these molecules are excessively produced in the body, they cause damage to cellular proteins, membrane lipids, and nucleic acids and reduce lifespan [99,100].

Several biomarkers have been widely considered predictors of oxidative stress; these include SOD, MDA, CAT, GPx, and GSH. The existing research suggests that *Abelmoschus esculentus* has antioxidant potential, partly due to its high phenolic and flavonoid content. For instance, two groups recently demonstrated a very high content of phenols and flavonoids in *Abelmoschus esculentus* mucilage and seed peel, respectively [101,102]. In contrast, other studies showed an increased half-maximal inhibitory concentration (IC50) in *Abelmoschus esculentus* extracts and mucilage compared to vitamin C [101,103]. This suggests the reduced antioxidant capability of *Abelmoschus esculentus*. IC50 refers to the number of antioxidant compounds necessary to scavenge 50% of the initial DPPH radicals. The compound’s increased effectiveness in scavenging DPPH radicals results in a reduced IC50 value, indicating an ideal level of antioxidant activity for the compound [104]. The summarized effect of *Abelmoschus esculentus* in rodent models of diabetes is presented in Table 2. Although the potential benefits are acknowledged, different studies have contradictory findings, with others showing negative results on oxidative stress markers. Below, we outline the effects of *Abelmoschus esculentus* on various markers of oxidative stress.

#### 4.1.2. *Abelmoschus esculentus* on Oxidative Stress with a Focus on ROS

In general, the evidence supports using *Abelmoschus esculentus* treatment as a possible antioxidant in at least three preclinical studies (Table 2). These studies revealed a significant (*p* < 0.05) reduction in the levels of ROS in a rodent model of DM and, therefore, an attenuation of oxidative stress [105,106,107]. Elevated ROS levels are associated with oxidative stress and organ damage [18]. Therefore, the potential of *Abelmoschus esculentus* to reduce these excess ROS may be of importance in reducing organ and tissue damage in diabetes mellitus. *Abelmoschus esculentus* potential to reduce ROS is associated with its high polyphenols, flavonoids, and vitamin C content as they scavenge free radical molecules, thus alleviating oxidative stress [79,80,81]. These compounds accomplish this activity by transferring hydrogen atoms to unstable ROS molecules, stabilizing them, and subsequently preventing any cell, tissue, or organ damage [108,109]. Similarly, *Abelmoschus esculentus* is rich in quercetin and catechin, which have antioxidant properties [74,75,76]. These compounds reduce NADPH oxidase activity, reducing ROS production and further oxidative stress [110,111].

#### 4.1.3. *Abelmoschus esculentus* on Oxidative Stress with a Focus on SOD

**Figure 4:**
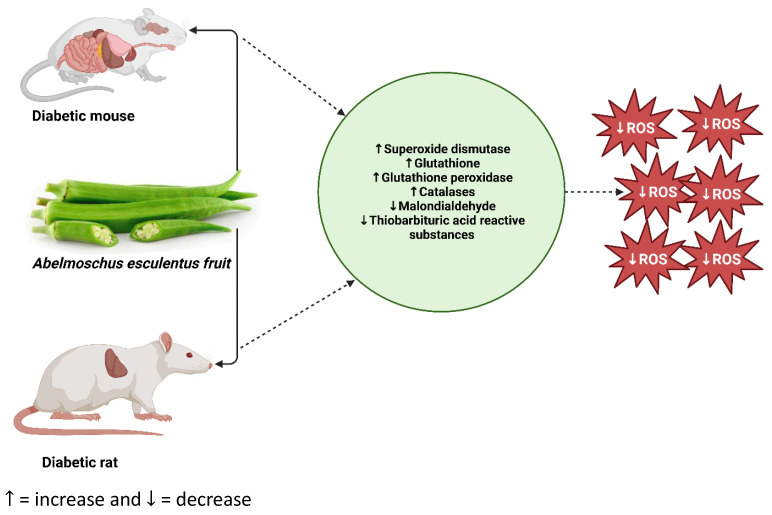
Potential mode of action of *Abelmoschus esculentus* in rodent models of diabetes. In a nutshell, oral administration of *Abelmoschus esculentus* ameliorates oxidative stress in rodent models of diabetes induced by either alloxan monohydrate or a high-fat diet coupled with streptozotocin. ROS: reactive oxygen species (https://prove.es/en/unknown-but-really-healthy-vegetable-called-okra/ (accessed on 25 July 2023)).

SOD is an antioxidant enzyme that protects cells against ROS if it is upregulated in the body [112]. In a model of DM, the central feature is oxidative stress, which exacerbates the condition. The administration of antioxidants, however, seems important as they reduce oxidative stress by increasing SOD levels. For example, as presented in Table 2, *Abelmoschus esculentus* is significantly (*p* < 0.05) associated with an increased SOD [96,106,113,114]. SOD is a crucial antioxidant that defends the body’s organs and cells from oxidative damage. Therefore, an increase in SOD in the body is important to help break down potentially harmful oxygen molecules in cells. An improvement of antioxidant status by *Abelmoschus esculentus* extract, as demonstrated by a significant increase in SOD, is commendable and thus may be relevant to attenuating oxidative stress (Figure 4). Such an effect of okra may further lead to ameliorating secondary complications associated with oxidative stress. However, other studies showed a significant (*p* < 0.05) decrease in SOD following the administration of *Abelmoschus esculentus* [91,102,115,116]. This suggests the limitation of *Abelmoschus esculentus* as an antioxidant; reduction of SOD in hyperglycemia or overproduction of ROS may subject the cells to damage and subsequently to apoptosis.

#### 4.1.4. *Abelmoschus esculentus* on Oxidative Stress with a Focus on CAT Activity

CAT is an active enzyme involved in the catalysis of hydrogen peroxide into water and oxygen [36]. However, due to reduced CAT activity, beta cells of the pancreas that contain many mitochondria undergo oxidative stress by producing excess ROS that leads to β-cells dysfunction and, ultimately, diabetes [18]. Interestingly, evidence presented in Table 2 showed that *Abelmoschus esculentus* treatment in rodent models of diabetes significantly increases CAT activity [91,95,105,106,113,115,117]. This suggests that *Abelmoschus esculentus* may ameliorate oxidative stress, further reduce complications of DM, or prevent DM in non-diabetics (Figure 4). Although the potential benefits of *Abelmoschus esculentus* on CAT activity have been noted in DM rodent models, another group of researchers has reported contradictory findings, as shown by significantly reduced CAT activity [116]. This, disappointingly, suggests a limited beneficial impact of *Abelmoschus esculentus* in improving the activity of CAT enzymes in diabetic models and, thus, its limited efficacy in reducing oxidative stress.

#### 4.1.5. Effects of *Abelmoschus esculentus* on Oxidative Stress: Focusing on MDA and TBARS

Additional oxidative stress markers include MDA, TBARS, lipid hydroperoxides (LH), and 4-hydroxy-2-Nonenal (4-HNE), the end products of lipid peroxidation. However, among these markers, MDA is widely studied and is regarded as an ideal marker of oxidative stress [118]. MDA seems to be elevated in DM, thus increasing the likelihood of developing complications associated with oxidative stress. It is assumed that the reduction of these markers can substantially alleviate oxidative stress and associated complications. Interestingly, preclinical evidence gathered in Table 2 showed *Abelmoschus esculentus* promising potential in reducing MDA amongst rodent models of DM [91,95,105,106,113,115,117]. Disappointingly, evidence from a preclinical model of GDM reported by Tian [113] showed different findings, which suggest the limitation of *Abelmoschus esculentus* in alleviating oxidative stress in GDM. Although the results were unfavorable, we believe this is due to insulin resistance occurring in the late stage of pregnancy and different pathophysiological mechanisms between GDM and DM, thus limiting the antioxidant potential of *Abelmoschus esculentus* [119]. Consistently, TBARS, a derivative of thiobarbituric acid and MDA, increases in response to oxidative stress [120]. Notably, an increased level of TBARS is observed in T1DM and T2DM, signifying oxalate toxicity induced by lipid peroxidation [121,122]. However, existing evidence showed a significant decrease in TBARS levels following *Abelmoschus esculentus* treatment in rodent models of DM [95,107]. Therefore, this reduction would suggest *Abelmoschus esculentus* potential for attenuating oxalate toxicity and a further reduction in oxidative stress.

**Table 2:** Overview of studies evaluating the antioxidant effect of *Abelmoschus esculentus* in a rodent model of diabetes mellitus.

Experimental Model	Treatment and Duration	Experimental Outcomes	Country	Reference
Alloxan monohydrate induced diabetes in Swiss albino female and male mice.	The suspension was prepared by dissolving the powdered peel seed (PPS) and *Abelmoschus esculentus* mucilage (PM) of *Abelmoschus esculentus* into distilled water. PPS and PM were administered orally at 150 and 200 mg/kg body weight for three weeks.	Total flavonoid content was higher in PPS than in PM. At the same time, the total phenol content was higher in PM than PPS. *Abelmoschus esculentus* had reduced antioxidant capabilities, as shown by a higher IC50 value in *Abelmoschus esculentus* PM and PPS compared to vitamin C.	Bangladesh	[101]
High-fat diet (HFD)-streptozotocin (STZ)-induced diabetes in SPF-grade C57BL/6 male mice.	*Abelmoschus esculentus* powder was isolated using distilled water and 80% ethanol precipitation from *Abelmoschus esculentus* and orally administered at 200 and 400 mg/kg of *Abelmoschus esculentus* powder for eight weeks.	*Abelmoschus esculentus* in diabetic mice reduced the level of reactive oxygen species (ROS) in diabetic mice compared to mice in the control group. A dose of 400 *Abelmoschus esculentus* powder in diabetic mice significantly increased superoxide dismutase (SOD), glutathione (GSH), and catalase (CAT) and decreased malondialdehyde (MDA) in the kidney when compared to the control group. The same pattern was observed at 200; however, this was not significantly different from diabetic controls.	China	[106]
HFD-fed-specific pathogen-free (SPF)-grade C57BL/6 male mice administered with STZ to induce diabetes.	*Abelmoschus esculentus* powder was dissolved in distilled water, and 200 or 400 mg/kg of body weight was orally administered for eight weeks.	*Abelmoschus esculentus* powder in diabetic mice decreased ROS and MDA and increased SOD, glutathione peroxidase (GPx), and CAT in the liver compared to the diabetic control. Nuclear factor erythroid 2–related factor 2 (Nrf2), heme oxygenase-1 (HO-1), and superoxide dismutase 2 (SOD2) expression were significantly upregulated by *Abelmoschus esculentus* powder.	China	[105]
Streptozotocin-induced diabetes in male Wistar albino rats.	*Abelmoschus esculentus* peel powder (AEPP) and *Abelmoschus esculentus* seed powder (AESP) were mixed with distilled water and administered orally at a dose of (100 or 200 mg/kg bw) for 28 days.	Administration of both doses of AEPP and AESP in diabetic rats significantly increased liver, kidney, and pancreatic SOD, CAT, GPx, and GSH levels compared to the diabetic controls.	India	[95]
STZ-induced diabetes in male Wistar rats.	*Abelmoschus esculentus* mucilage extract and *Abelmoschus esculentus* seed aqueous solution were made by dissolving the powder in ethanol. The extract was orally administered at 150 and 200 mg/kg of body weight for 30 days.	*Abelmoschus esculentus* in diabetic rats significantly decreased MDA and increased catalase, SOD, and GSH activity compared to diabetic controls.	Saudi Arabia	[115]
STZ-induced diabetes in male Wistar rats.	About 50 g of *Abelmoschus esculentus* powder was used to make the following extracts: (ethanolic extract 75%, ethanolic extract 90%, aqueous extract, and ethyl acetate extract). *Abelmoschus esculentus* extracts were orally administered at 200 and 400 mg/kg doses for eight weeks.	*Abelmoschus esculentus* extracts had reduced antioxidant properties, as revealed by an increased half-maximal inhibitory concentration (IC50) level compared to vitamin C and quercetin.	Iran	[103]
STZ-induced diabetes mellitus in adult Wistar rats.	*Abelmoschus esculentus* powder was mixed with food and given as a food pellet to the rats.	*Abelmoschus esculentus*-mixed diets in diabetic rats significantly reduced SOD, CAT, GSH, MDA, and α-amylase. However, GPx in *Abelmoschus esculentus* was not significantly different from diabetic controls.	Nigeria	[116]
Alloxan-induced diabetes in female and male Wistar rats.	Whole *Abelmoschus esculentus* (WAE), *Abelmoschus esculentus* peel (AEP), and *Abelmoschus esculentus* seed (AES). *Abelmoschus esculentus* samples (WAE, AEP, and AES) were administered at 100, 200, and 300 mg/kg by single forced oral feeding once daily for 21 days.	*Abelmoschus esculentus* in diabetic rats significantly increased CAT activity and GSH and decreased MDA compared to diabetic controls.	Nigeria	[117]
HFD fed Spraque–Dawley male rats were injected with STZ to induce diabetes.	*Abelmoschus esculentus* subfractions (F1, F2, and FR), the ethanol-extracted subfraction, and distilled water residue of *Abelmoschus esculentus* oral feed for 12 weeks.	*Abelmoschus esculentus* in diabetic rats has benefits in regulating dipeptidyl peptidase-4 (DPP-4) and the glucagon-like peptide 1 receptor (GLP-1R), thus reducing oxidative stress. *Abelmoschus esculentus* in diabetic rats significantly decreased serum and kidney TBARS compared to diabetic controls. All extracts lowered peroxidation except for fraction 1.	Taiwan	[107]
Alloxan-induced diabetes in male Wistar strain rats.	*Abelmoschus esculentus* powder (2 g/kg rat body weight) was mixed well in 0.2% carboxy methyl cellulose (CMC) and fed to animals by gavage technique at 2 g/kg body weight for 35 days.	*Abelmoschus esculentus* in diabetic rats significantly increased lipid peroxidation in erythrocytes, GSH, and decreased MDA in the kidney compared to diabetic controls.	India	[123]
Alloxan monohydrate induced diabetes in male and female Wistar rats.	The animal diet was prepared by mixing 33 g of the *Abelmoschus esculentus* powder with 67 g of normal rat feed, and 66 g of *Abelmoschus esculentus* was mixed with 34 g of normal rat feed to obtain the 33% and 66% supplement ratios and fed to rats for 16 days.	*Abelmoschus esculentus* powder in diabetic rats significantly decreased SOD and MDA levels and increased GSH and CAT activity compared to diabetic controls.	Nigeria	[91]
STZ-induced diabetes in male Sprague–Dawley rats.	The *Abelmoschus esculentus* powder samples (250 g) were subjected to extraction procedures using ten volumes of either 75% ethanol or distilled water. 100 mL/kg of solution (aqueous extract, ethanol extract, and aqueous extract of Indole Acetic Acid (IAA) from *Abelmoschus esculentus*) was administered orally daily for six weeks.	*Abelmoschus esculentus* powder significantly decreased SOD in diabetic rats that received ethanol extract compared to diabetic controls. Diabetic rats that received an aqueous extract of *Abelmoschus esculentus* at 100 mg/kg had significantly increased liver total phenolic content.	Canada	[102]
STZ-induced hyperglycemia in male Wistar rats.	Three fresh *Abelmoschus esculentus* pods were sliced and infused in 250 mL of 3.6 mL *Abelmoschus esculentus* infusion water for 28 days.	*Abelmoschus esculentus* in diabetic rats significantly increased SOD when compared to diabetic controls.	Bangladesh	[114]
STZ-induced gestational diabetes (GDM) in Spraque–Dawley rats.	The intervention group was administered orally a solution containing 200 mg/kg of *Abelmoschus esculentus* extract.	*Abelmoschus esculentus* in GDM rats significantly increased SOD, MDA, CAT, GSH, and GPx in the liver and pancreas compared to GDM controls.	China	[113]

SOD: superoxide dismutase, MDA: malondialdehyde, CAT: catalase, GSH: glutathione, GPx: glutathione peroxidase, CMC: carboxy methyl cellulose, IAA-AE: Indole Acetic Acid-*Abelmoschus esculentus*, AEPP: *Abelmoschus esculentus* peel powder, AESP: *Abelmoschus esculentus* seed powder, GDM: gestational diabetes, F1: fraction 1, FR: fractional residue, DPP-4: dipeptidyl peptidase-4, GLP-1R: glucagon-like peptide 1 receptor, WAE: whole *Abelmoschus esculentus*, AEP: *Abelmoschus esculentus* peel, AES: *Abelmoschus esculentus* seed, SPF: specific pathogen-free, IC50: half maximal inhibitory concentration, TT: total thiols, TBARS: thiobarbituric acid reactive substances, Nrf2: Nuclear factor erythroid 2–related factor 2.

#### 4.1.6. *Abelmoschus esculentus* on Glutathione and Glutathione Peroxidase

GSH is a group of enzymes that protect the body from oxidative stress by reducing lipid hydroperoxides and free hydrogen peroxide [124,125]. The general overview of the effect of *Abelmoschus esculentus* on markers of GPx and GSH is outlined in Table 2.

However, a decrease in its activity subjects the cells or organs to oxidative stress by allowing the accumulation of harmful oxidants and free radicals. Therefore, antioxidant compounds that increase the activity of GSH and GPx can be used as an alternative therapy to ameliorate oxidative stress and protect the cells from oxidative damage (Figure 4). Our current review found contradictory reports from various rodent models of DM induced by STZ or alloxan monohydrate. Of interest was that several studies reported a significant increase in the activity of GSH [91,95,105,106,115,123]. Additionally, Tian et al. [113] and Aleisa et al. [115] have reported a noteworthy augmentation in the activity of GPx, further substantiating the potential of *Abelmoschus esculentus* and its constituents as antioxidant agents.

In contrast to the aforementioned encouraging discoveries, other researchers [91,95,105,106,115,116,123] have recently identified a notable decline in GSH levels after administering *Abelmoschus esculentus* treatment to rodent models with diabetes. This once again highlights a potential limitation of *Abelmoschus esculentus* as an antioxidant. *Abelmoschus esculentus* ability to regulate oxidative stress seems to be attributable to its high content of phenols, flavonoids, and associated minerals, as presented in Figure 3.

## 5. Limitations

The current review has several limitations; for instance, the evidence gathered here is primarily from preclinical studies, with mainly mice and rats used for such experimentation. Additionally, the majority of models of diabetes presented here were primarily developed by using the administration of either STZ or alloxan monohydrate, with a few using HFD to induce diabetes. It is commonly known that these two drugs cause pancreatic damage, and the model developed through their administration mimics that of T1DM, while HFD resembles that of T2DM seen in humans. Since the pathogenesis of these conditions differs, the interpretation may be skewed toward T1DM, as most studies have induced diabetes through drug intervention. While evidence from the preclinical studies supports the use of both *Amaranthus* and *Abelmoschus esculentus* as herbal treatments for DM against oxidative stress, it is still not clear as to which part of these plants is more beneficial, as some studies used leaves, seeds, and pods.

The exact dose or form by which these plants are administered is also not specified, as a powder has been used to prepare the extract or given to rodents as a food-powder mixture. Although other clinical studies have been conducted on *Abelmoschus esculentus* in diabetic populations, ranging from randomized controlled trials to quasi-experiments, such studies did not focus on oxidative stress or related markers [126]. Moreover, regarding *Amaranthus*, only one trial has been conducted in diabetic patients, and the results are promising [127]. However, since then, clinical evidence has been scarce. Lastly, Amaranthus, and *Abelmoschus esculentus* have been proven effective in reducing diabetes. However, to the best of our knowledge, no studies have been conducted using a combined treatment of both of these plants in diabetic models or clinical trials. As the evidence reviewed here is derived from preclinical studies, it is important to note that in some cases, the evidence from experimental studies is not fully translatable to humans due to different physiological systems and functions.

## 6. Conclusions and Recommendations

The study discussed the effects of different parts of *Amaranthus* and *Abelmoschus esculentus*, highlighting their potential as alternative remedies to attenuate oxidative stress in diabetes models. Current evidence on the recommended dosages for these plants has proven these plants to be considered safe in the management of DM. Both plants are rich in carbohydrates, proteins, fatty acids, vitamins, fiber, minerals, and other bioactive phytochemicals that promote good health and are less expensive, making them economically affordable natural antioxidants. Evidence exploring the efficacy and safety of *Abelmoschus esculentus* in diabetes on other markers exists [17,101], but studies in this population still lack exploration of oxidative stress. While the potential benefits of *Amaranthus* have been extensively explored in diabetic models, there is limited evidence in clinical trials, especially on oxidative stress. The preclinical evidence gathered in this review revealed that *Amaranthus* treatment in diabetes could ameliorate hyperglycemic-associated oxidative stress (Figure 2). Remarkably, our summarized evidence also demonstrated that the administration of *Abelmoschus esculentus* to diabetic rodents also attenuates oxidative stress (Figure 4). Although there have been limited clinical trials involving *Abelmoschus esculentus* in diabetic populations [126], its potential benefits are widely recognized within the broader research community, and its safety has been confirmed. However, since there are currently no trials investigating the effects of these plants on oxidative stress, it is recommended that future trials be conducted, especially in African countries where the prevalence of DM is high. Therefore, we plan to explore oxidative stress with well-designed and adequately powered clinical studies. Therefore, future studies may be necessary to fully understand its efficacy, optimal dosage, and impact on oxidative stress. These studies should be well-designed and adequately powered, focusing on diabetic patients.

## Figures and Tables

**Table 1:** Overview of studies evaluating the antioxidant effect of *Amaranthus* rodents in a model of diabetes mellitus.

Experimental Model	Treatment and Duration	Experimental Outcomes	Country	Reference
Alloxan-induced diabetic albino Wistar rats.	Methanol extract of *Amaranthus spinosus* (MEAS) leaves. Oral administration of MEAS (200 and 400 mg/kg) for 15 days.	*Amaranthus spinosus* in diabetic rats significantly reduced malondialdehyde (MDA) and increased glutathione (GSH), catalase (CAT), and total thiols (TT) compared to diabetic control.	India	[65]
Streptozotocin (STZ)-induced diabetes in Wistar rats.	Ethanolic extract of *Amaranthus spinosus* leaves. Intraperitoneal injection of *Amaranthus* at doses (250 and 500 mg/kg) for 21 days.	*Amaranthus spinosus* in diabetic rats significantly increased GSH, superoxide dismutase (SOD), CAT, and glutathione peroxidase (GPx) compared to the diabetic control.	India	[66]
STZ-induced diabetic albino Wistar rats.	Ethanol extract of *Amaranthus hybridus* leaves. Oral administration of AHELE at doses (200 and 400 mg/kg) for 14 days.	*Amaranthus hybridus* in diabetic rats significantly reduced thiobarbituric acid reactive substances (TBARS) and increased SOD and CAT activity compared to diabetic control.	India	[67]

MEAS: methanol extract of *Amaranthus spinosus*, STZ: streptozotocin, SOD: superoxide dismutase, MDA: malondialdehyde, CAT: catalase, GSH: glutathione, GPx: glutathione peroxidase, IC50: inhibitory concentration 50, TT: total thiols, TBARS: thiobarbituric acid reactive substances, AHELE: *Amaranthus hybridus* ethanol leaf extract.

## Data Availability

Not applicable.

## Abbreviations

DM: Diabetes mellitus; T2DM: Type 2 diabetes mellitus; T1DM: Type 1 diabetes mellitus; AHELE: *Amaranthus hybridus* ethanol leaf extract; CAT: Catalase; GSH: Glutathione; GPx: Glutathione peroxidase; SOD: Superoxide dismutase; MDA: Malondialdehyde; TBARS: Thiobarbituric acid reactive substances; and STZ: Streptozotocin; IAA: Indole Acetic Acid; PM: Powdered nucillage; PPS: powdered peel seed; ROS: Reactive oxygen species; AEP: *Abelmoschus esculentus* powder; HFD: High-fat diet; AEPP: *Abelmoschus escuelentus* Peel powder; AESP: *Abelmoschus esculentus* seed powder; DPPH: 1,1-diphenyl-2-picrylhydrazyl; ABTS: 2,2′-casino-bis-3-ethylbenzothiazoline-6-sulfonic acid; LMICs: low- and middle-income countries; FFA: Free fatty acids; AGEs: advanced glycation end products; mNADH: mitochondrial NADH; DAG: diacylglycerol; PKC: Protein kinase C; ET-1: endothelin 1; VCAM-1: vascular cell adhesion molecule; ICAM-1: intercellular adhesion molecule; NF-κβ: nuclear factor kappa-light-chain-enhancer of activated β cell; CMC: Carboxy methyl cellulose; GDM: gestational diabetes; F1: Fraction 1; FR: Fractional residue; GLP-1R: Glucagon like peptide 1 receptor; WAE: Whole *Abelmoschus esculentus*; SPF: Specific pathogen-free.
